# Non‐enzymatic glycation reduces glucose transport in the human cartilage endplate independently of matrix porosity or proteoglycan content

**DOI:** 10.1002/jsp2.1297

**Published:** 2023-10-24

**Authors:** Jae‐Young Jung, Mohamed Habib, Luke J. Morrissette, Shannon C. Timmons, Tristan Maerz, Aaron J. Fields

**Affiliations:** ^1^ Department of Orthopaedic Surgery University of California San Francisco San Francisco California USA; ^2^ Department of Natural Sciences Lawrence Technological University Southfield Michigan USA; ^3^ Departments of Orthopaedic Surgery and Biomedical Engineering University of Michigan Ann Arbor Michigan USA

**Keywords:** advanced glycation end products, CA^4+^, cartilage endplate, disc degeneration, glucose uptake, low back pain, matrix crosslinks

## Abstract

**Background:**

Intervertebral disc degeneration is associated with low back pain, which is a leading cause of disability. While the precise causes of disc degeneration are unknown, inadequate nutrient and metabolite transport through the cartilage endplate (CEP) may be one important factor. Prior work shows that CEP transport properties depend on the porosity of the CEP matrix, but little is known about the role of CEP characteristics that could influence transport properties independently from porosity. Here, we show that CEP transport properties depend on the extent of non‐enzymatic glycation of the CEP matrix.

**Methods and Results:**

Using in vitro ribosylation to induce non‐enzymatic glycation and promote the formation of advanced glycation end products, we found that ribosylation reduced glucose partition coefficients in human cadaveric lumbar CEP tissues by 10.7%, on average, compared with donor‐ and site‐matched CEP tissues that did not undergo ribosylation (*p* = 0.04). These reductions in glucose uptake were observed in the absence of differences in CEP porosity (*p* = 0.89) or in the amounts of sulfated glycosaminoglycans (sGAGs, *p* = 0.47) or collagen (*p* = 0.61). To investigate whether ribosylation altered electrostatic interactions between fixed charges on the sGAG molecules and the mobile free ions, we measured the charge density in the CEP matrix using equilibrium partitioning of a cationic contrast agent using micro‐computed tomography. After contrast enhancement, mean X‐ray attenuation was 11.9% lower in the CEP tissues that had undergone ribosylation (*p* = 0.02), implying the CEP matrix was less negatively charged.

**Conclusions:**

Taken together, these findings indicate that non‐enzymatic glycation negatively impacts glucose transport in the CEP independent of matrix porosity or sGAG content and that the effects may be mediated by alterations to matrix charge density.

## Graphical abstract

Inadequate nutrient and metabolite transport through the cartilage endplate (CEP) may be one important factor in the etiology of disc degeneration. Prior work shows that CEP transport properties depend on the porosity of the CEP matrix, but little is known about the role of CEP characteristics that could influence transport properties independently from porosity. This study demonstrates that non‐enzymatic glycation negatively impacts glucose transport in the CEP independent of matrix porosity or sGAG content and that the effects may be mediated by alterations to matrix charge density.

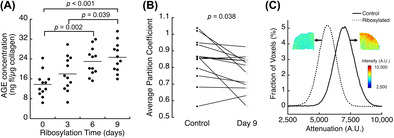

## 1 INTRODUCTION

Low back pain (LBP) is the leading cause of disability, and in certain subgroups, LBP may be caused by intervertebral disc degeneration.^1^ Although the precise causes of disc degeneration are unclear, poor disc nutrition is believed to be an important factor. The cartilage endplate (CEP) plays an important role in disc nutrition because the cells within the avascular nucleus pulposus rely on nutrients and metabolites that must pass through the CEP. For example, low CEP permeability hinders the survival and function of nucleus pulposus cells in vitro^2^ and is associated with more severe disc degeneration in vivo.^3^, ^4^ Elucidating the characteristics of the CEP matrix that inhibit solute transport could improve our understanding of the mechanisms of disc degeneration and help guide the development of diagnostic tools and therapeutic strategies for disc regeneration.

Several characteristics of the CEP matrix influence its transport properties. The overall porosity of the CEP matrix is an important determinant of solute transport because porosity governs the amount of pore space available to the solutes. For example, glucose partition coefficients and diffusivities in the CEP, which reflect the maximum concentration of glucose that can enter the CEP and the rate of diffusion through the CEP matrix, are negatively correlated with proteoglycan content^5^ and positively correlated with CEP porosity.^6^, ^7^, ^8^

Although much is known about the roles of porosity and matrix content, less is known about matrix characteristics that could influence solute transport that are independent of porosity or matrix content. One such characteristic is the degree of non‐enzymatic cross‐linking. Non‐enzymatic cross‐links (so‐called “advanced glycation end products” or AGEs) increase in the disc with aging and with disc degeneration.^9^ AGEs form via non‐enzymatic glycation of the free amino groups of matrix proteins such as collagen and aggrecan by the reduction of sugars, and they accumulate readily because of the slow turnover of the disc matrix. Separately, AGE accumulation appears to be accelerated by a Western diet^10^ and by hyperglycemia.^11^, ^12^ The formation of AGEs leads to protein aggregation^13^ and collagen structure alteration,^14^ which can increase the propensity for collagen damage in the annulus fibrosus and lead to early disc degeneration.^15^, ^16^ Additionally, AGEs change the molecular charge density and charge distribution of the matrix.^11^, ^17^ In theory, such changes could impact solute transport through the CEP, although the precise nature and extent of these effects are unknown.

The goal of this study was to determine how AGE accumulation in human CEP tissue impacts biochemical composition, porosity, matrix charge distribution, and glucose transport. We hypothesized that AGE accumulation reduces glucose uptake and that the effect of AGEs on glucose uptake is independent of matrix porosity and coincides with reductions in the net hydrophilic charge of the CEP. To characterize the fixed charge distribution within the CEP, we performed equilibrium partitioning of the cationic contrast agent CA^4+^ by micro‐computed tomography (micro‐CT).^18^, ^19^, ^20^ This study is unique because it is the first to measure the effects of AGEs on solute transport in the CEP, and it is the first to measure the effects of AGEs on matrix charge distribution using contrast‐enhanced micro‐CT.

## 2 MATERIALS AND METHODS

### 2.1 Human cadaver imaging and CEP tissue harvesting

Twelve fresh cadaveric lumbar spines (age range: 25–73 years; average: 55.6 ± 12.8 years; eight males and four females) were obtained <72 h postmortem from donors with no medical history of musculoskeletal disorders (UCSF Willed Body Program). Prior to spine harvest, cadavers underwent magnetic resonance imaging (GE Discovery MR 750W 3T; GE Healthcare), which consisted of a sagittal fast spin‐echo T2‐weighted sequence with the following parameters: echo‐time 61.6 ms; repetition time 2500 ms; echo train length 8; acquisition matrix 512 × 512; slice thickness 3 mm; and field‐of‐view 27 cm × 27 cm. Disc degeneration was evaluated using the Pfirrmann classification system.^4^, ^21^

After magnetic resonance imaging, intact CEPs from the central region of the L4‐L5 and L5‐S1 discs were removed from the subchondral bone and trimmed of nucleus pulposus tissue with a razor blade. All CEP samples were free from obvious degenerative pathology (e.g., fissures and calcification) and were stored in saline‐soaked gauze at −80°C and thawed before subsequent experiments. For every level and donor, the harvested CEP tissues were prepared into circular samples using a biopsy punch (4 mm‐diameter). Six to eight site‐matched CEP samples were prepared per spinal level for each donor (Table 1); the CEP samples were harvested from either the superior endplate (five donors) or inferior endplate (seven donors). Samples harvested from the inferior endplates were pooled with samples harvested from the superior endplates. In total, 104 full‐thickness CEP samples were used for this study. The thickness of the CEP samples was measured with a micrometer designed to sense the tissue's electrical conductivity.^22^

**TABLE 1:** Summary of donor age, sex, disc level, endplate location, and Pfirrmann grade.

Age	Sex	Disc level	Endplate location	Pfirrmann grade
25	M	L5‐S1	Inferior	III
38	M	L4‐L5	Superior	III
52	M	L5‐S1	Superior	III
55	M	L4‐L5	Inferior	III
56	M	L5‐S1	Inferior	III
57	M	L4‐L5	Superior	IV
60	F	L4‐L5	Superior	III
61	M	L5‐S1	Inferior	III
63	F	L5‐S1	Inferior	III
63	F	L4‐L5	Superior	IV
64	M	L5‐S1	Inferior	IV
73	F	L4‐L5	Inferior	III

### 2.2 In vitro ribosylation

To induce the formation of AGEs, we used an in vitro ribosylation procedure that was modified from previous studies.^23^, ^24^ Briefly, the CEP tissues were incubated in ribose solution (0.6 M in Hank's balanced salt solution, HBSS) with protease inhibitors. We used HBSS containing phenol red (40 μM) to monitor pH levels; the ribose solution was adjusted to pH 6.8–7.2 every 2 days during the designated incubation period. After in vitro ribosylation, the CEP samples were rinsed with phosphate‐buffered saline (PBS). One sample from each donor was allocated into each of four groups for the biochemistry studies: 0 (control), 3, 6, and 9 days incubation time (*n* = 12 samples/group). An additional four CEP samples from each donor were used for the glucose uptake studies and included two CEP samples/donor per group for each of two groups: 0 (control) and 9 days incubation time. An additional two CEP samples from four donors were used for the contrast‐enhanced micro‐CT experiments and included one CEP sample/donor per group for each of two groups: 0 (control) and 9 days incubation time. Due to the incompatibility of the glucose uptake, biochemistry, and micro‐CT protocols, it was not possible to perform all of the measurements on the same CEP samples.

### 2.3 Glucose uptake

To measure glucose uptake in the CEP, we used a two‐bath protocol^25^ for measuring the equilibrium partition coefficient. The equilibrium partition coefficient, which is defined as the equilibrium concentration of a solute in the matrix relative to that in the surrounding solution, is an important characteristic that influences solute transport through the tissue. In the two‐bath protocol, CEP samples were first immersed in a glucose absorption bath (3.33 mg/mL D‐(+)‐glucose in PBS; G8270; Sigma Aldrich) for 24 h at 4°C with constant mild agitation. Following glucose absorption, the samples were removed, blotted dry with a tissue, and transferred to the desorption bath (PBS only) for 24 h at 4°C. The concentration of glucose in each bath at the end of the 24 h absorption and desorption periods was determined using glucose assay reagent (G3293; Sigma Aldrich). An aliquot from each bath was incubated with the glucose assay reagent for 15 min at room temperature, the absorbance of the aliquots was measured at 340 nm with a spectrophotometer, and glucose concentration was interpolated from a standard curve with known glucose concentrations. The partition coefficient, *K*, which is defined by *c* = *Kc*_*b*_ where *c* is the solute concentration in the sample and *c*_*b*_ is the solute concentration in the surrounding solution, was then determined by assuming conservation of solute between the absorption bath and the desorption bath^25^:

$$K=\frac{c_{2}v_{b2}}{\phi^{w}v\;\left(c_{1}-c_{2}\right)}$$

where $v_{b2}$ was the volume of the desorption bath, vwas the volume of the CEP sample, $\phi^{w}$ was the porosity of the CEP sample (see below), and $c_{1}$and $c_{2}$ were the concentrations of the absorption and desorption baths at equilibrium.

### 2.4 CEP porosity and biochemistry

CEP porosity ($\phi^{w}$) was determined using the buoyancy method.^6^, ^26^ The wet weight in air and the submerged weight in PBS were measured using a semi‐microanalytical balance fitted with a density kit. Finally, the CEP samples were lyophilized to measure dry weight, digested in papain, and assayed for sulfated glycosaminoglycan (sGAG) content (1,9‐dimethylmethylene blue assay^27^), collagen content (Chloramine T assay^28^), and total AGE content (fluorimetric assay with quinine sulfate standard^24^).

### 2.5 Contrast‐enhanced micro‐computed tomography

Contrast‐enhanced micro‐CT via equilibrium partitioning of an ionic contrast agent is a nondestructive technique used to estimate sGAG content in cartilaginous tissues. Owing to the net negative charge of the sGAGs, immersion of cartilage and disc tissues in the cationic contrast agent CA^4+^ results in uptake of the positively charged contrast agent, and inter‐sample differences in the measured micro‐CT attenuation are positively correlated with inter‐sample differences in the mean sGAG content.^29^ Here, we used the iodinated cationic contrast agent CA^4+^,^19^, ^20^, ^29^, ^30^ which was synthesized as described previously.^19^, ^20^ Four site‐ and donor‐matched pairs of CEP samples were incubated in the ribose solution (one sample from each pair for 0 days [control] and one for 9 days) and then scanned at baseline with a micro‐CT scanner (Scanco, μCT50). Scans were performed with a tube voltage of 50 kVp, a current of 114 mA, an integration time of 250 ms, and an isotropic voxel size of 12 μm.

Samples were then immersed in 15 mg I/mL CA^4+^ solution and imaged with micro‐CT after 0.5, 1, 2, 4, 12, and 24 h of incubation. Following CA^4+^ absorption, CEP samples were transferred to PBS for desorption and imaged with micro‐CT after 1, 12, and 24 h. Reconstructed images were postprocessed using a custom script in IDL (v8.1; L3Harris Geospatial) to measure the mean X‐ray attenuation and its standard deviation within a region of interest (ROI) placed inside the center of each sample. Each cylindrical ROI had a diameter of 504 μm (equivalent to 42 voxels) and height equivalent to the full height of the sample. Prior to measuring the X‐ray attenuation in each ROI, a constant threshold value (1983) was chosen to select the minimum intensity of the sample without a contrast agent, and that threshold value was applied to the raw micro‐CT data to help delineate the sample boundaries for ROI selection. The mean X‐ray attenuation inside the ROIs was compared between CEP samples in the ribosylated and control groups for each time point in a paired manner since every sample incubated in ribose solution for 9 days had a matched control sample from the same donor and level incubated in HBSS without ribose.

### 2.6 Statistical analysis

A one‐way analysis of variance (ANOVA) with Tukey–Kramer post hoc tests was used to determine the effects of ribosylation time on the various outcomes. A matched pair *t*‐test was used to compare the average partition coefficient and X‐ray attenuation between two groups of donor and site‐matched CEP samples (control vs. day 9 ribosylated). Linear regression was used to determine the independent correlations between the partition coefficient (outcome) and CEP porosity and hydration (explanatory variables) and between the ribosylation‐induced changes in partition coefficient (outcome) and average CEP porosity and hydration (explanatory variables). We used parametric tests because the data did not violate the normality assumption (*p* > 0.05, Shapiro–Wilk test) or equal variance assumption. All analyses including mean difference and 95% CI were calculated using JMP 16 Pro (SAS Institute; Cary, NC). Significance was defined as *p* < 0.05. Data are given as mean ± standard deviation.

## 3 RESULTS

**FIGURE 1:**
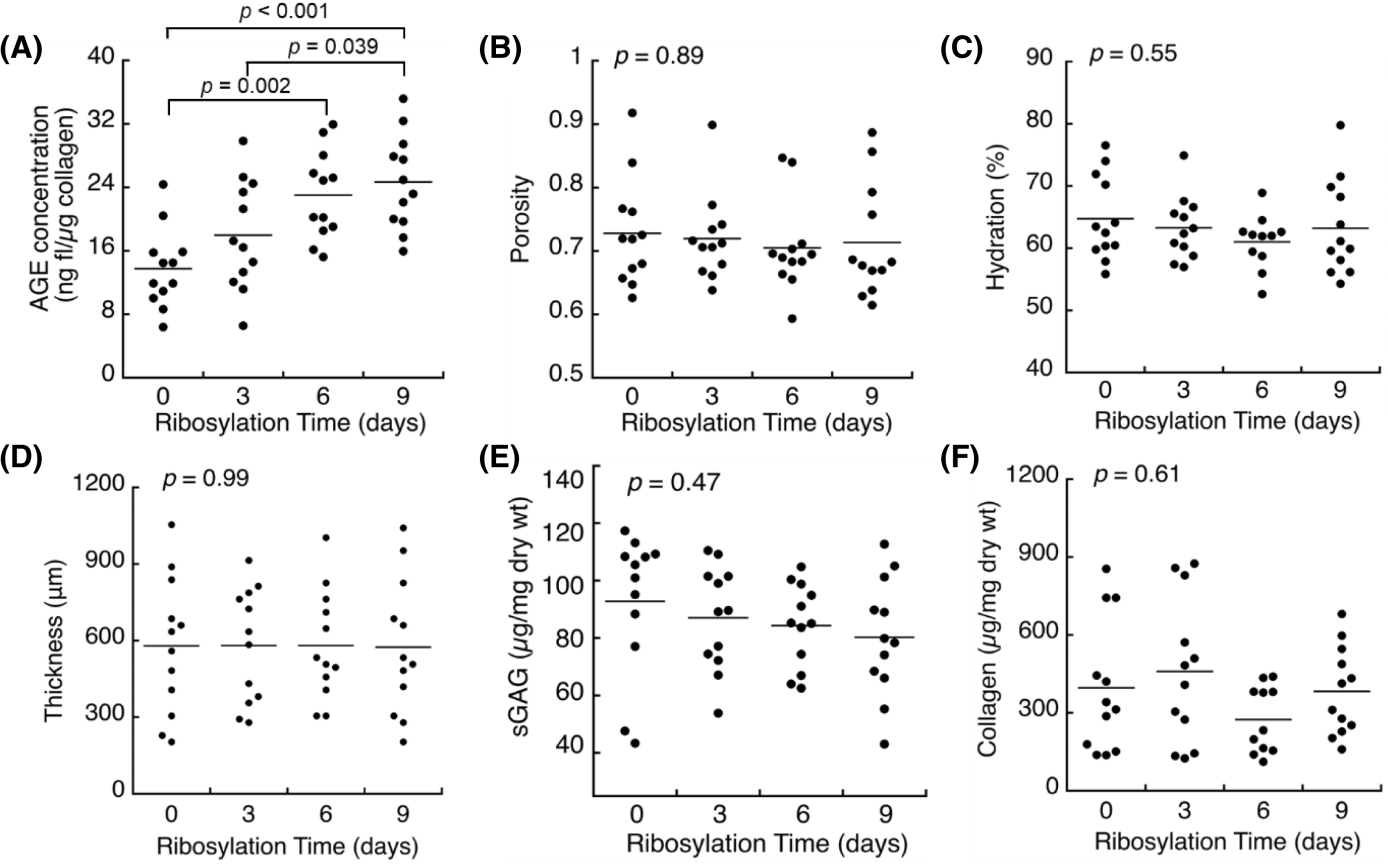
Human CEP samples were treated for 0–9 days with 0.6 M ribose solution. Ribosylation significantly increased AGE concentration (A) and had no statistically significant effects on bulk porosity (B), hydration (C), thickness (D), sGAG content (E), or collagen content (F). *n* = 11–12 samples/group. *p*‐values from one‐way ANOVA with Tukey post hoc test; all other comparisons are not significant. AGE, advanced glycation end product; ANOVA, analysis of variance; CEP, cartilage endplate; sGAG, sulfated glycosaminoglycan.

Ribosylation significantly increased the concentration of AGEs in the CEP in a time‐dependent manner (*p* < 0.0001; Figure 1A). Compared to the control group (day 0), ribosylated samples had AGE concentrations that were 131% (day 3), 167% (day 6), and 179% (day 9) higher, on average. At the same time, there were no statistically significant effects of ribosylation time on bulk CEP porosity (*p* = 0.89; Figure 1B), hydration (*p* = 0.55; Figure 1C), thickness (*p* = 0.99; Figure 1D), sGAG content (*p* = 0.47; Figure 1E), or collagen content (*p* = 0.61; Figure 1F). The mean CEP thickness for all samples was 578.3 ± 236.4 μm, and there was no difference in mean CEP thickness among the four ribosylation groups (0 [control], 3, 6, and 9 days: ANOVA *p* = 0.99, *n* = 12 samples/group).

**FIGURE 2:**
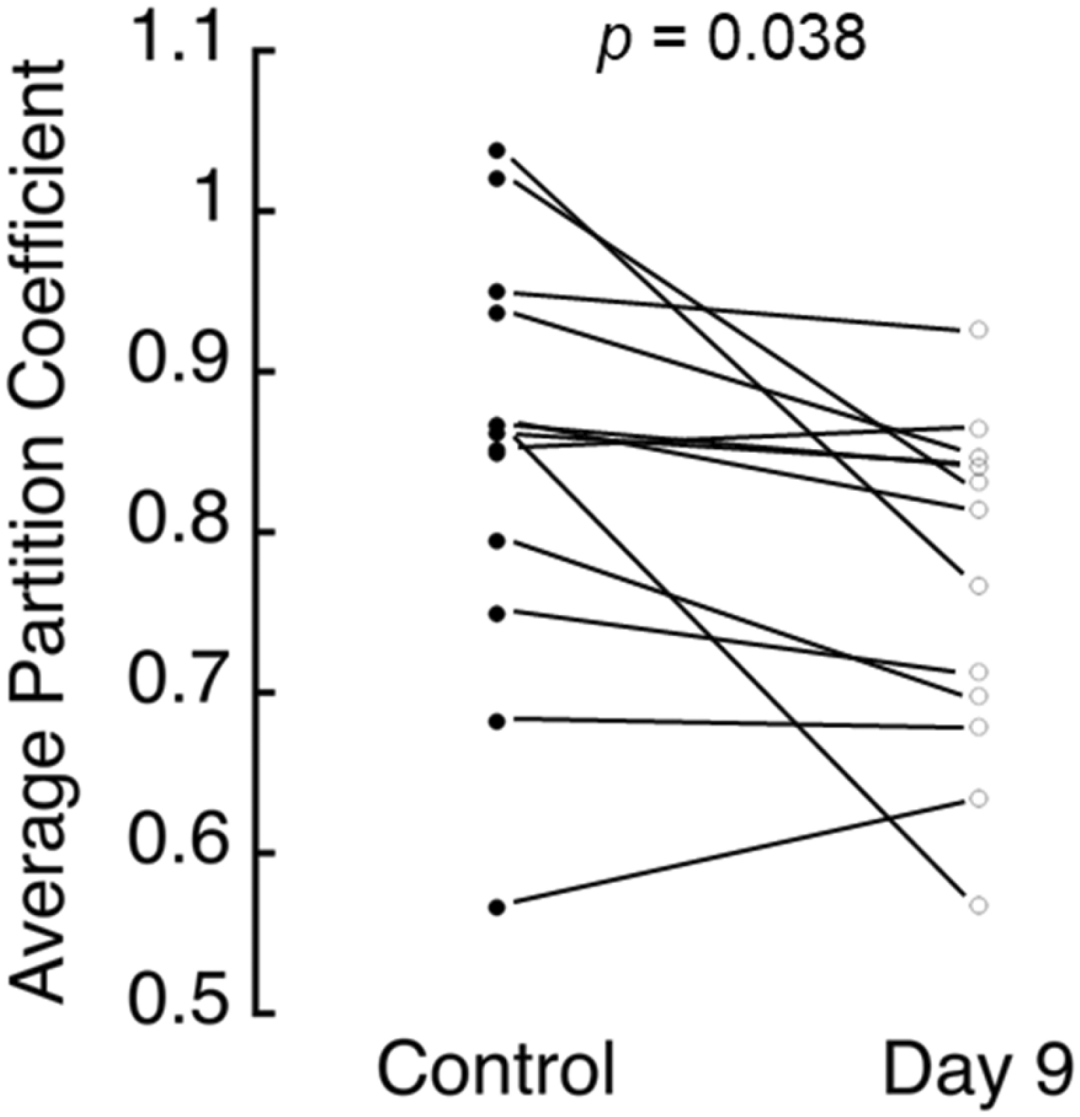
Average partition coefficients for glucose (180 Da) in the CEP were significantly lower in the ribosylated group (0.77 ± 0.11) than in the control group (0.85 ± 0.14) by paired *t‐*test. Each data point represents the average partition coefficient for two samples from each donor and site. The mean difference (control minus day 9) was 0.082 (95% CI, 0.0112–0.1528). CEP, cartilage endplate.

To understand whether AGE accumulation induced by in vitro ribosylation negatively impacted solute transport in the CEP, we measured glucose uptake during equilibrium partitioning experiments. As shown in Figure 2, ribosylation time had a small but significant effect on glucose uptake; on average, partition coefficients were 10.7% lower in ribosylated samples compared with their donor‐ and site‐matched controls (*p* = 0.038; paired *t‐*test). In general, partition coefficients were positively associated with CEP porosity (*r* ^2^ = 0.24, *p* = 0.0005, Figure S2) and with CEP hydration (*r* ^2^ = 0.24, *p* = 0.0004). Ribosylation‐induced changes in glucose partition coefficients were not correlated with CEP porosity (*p* = 0.85) or CEP hydration (*p* = 0.81), indicating the effects of ribosylation on glucose partition coefficient did not depend on CEP porosity or hydration.

**FIGURE 3:**
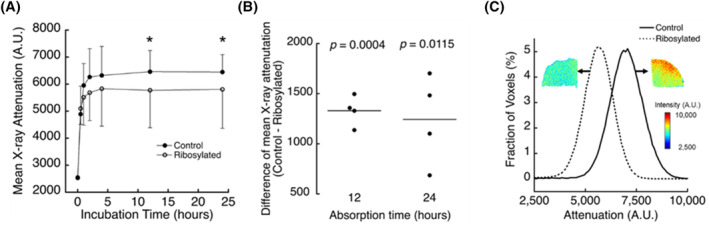
(A) Mean X‐ray attenuation of CEP samples incubated in ribose solution for 0 days (control) and 9 days (ribosylated) during CA^4+^ absorption. *p < 0.05 by paired *t*‐test. (B) Mean X‐ray attenuation was significantly lower in the ribosylated group after 12 and 24 h of absorption. Control minus ribosylated; differences >0 indicate higher value in control sample than paired ribosylated sample. The mean difference between groups (control minus ribosylated) after 12 and 24 h of absorption was significantly different from zero: 12 h, 1329.70 (95% CI, 1094.7–1564.70; *p* = 0.0004); 24 h, 1242.40 (95% CI 531.31–1953.50, *p* = 0.0115). (C) Representative 2D images and histograms of X‐ray attenuation from a control (day 0) and ribosylated (day 9) sample after contrast agent absorption for 24 h. The histograms show the statistical distribution of attenuation values from the 3D regions‐of‐interest that were used to calculate the mean attenuation. CEP, cartilage endplate.

To determine how AGE accumulation altered the net electrostatic charge of the CEP matrix, we immersed matched pairs of CEP samples in CA^4+^ and assessed the kinetics of contrast agent absorption by micro‐CT (Figure 3). Ribosylation significantly lowered time‐dependent CA^4+^ uptake in the CEP, and samples incubated in ribose for 9 days had 11.9% lower X‐ray attenuation at equilibrium (12 and 24 h), on average (Figure 3A,B). In general, histograms of X‐ray attenuation values from control and ribosylated samples had similar shapes as the control samples but with the histogram peak shifted to the left (Figure 3C).

## 4 DISCUSSION

Glucose transport through the CEP is critical for NP cell survival and function,^2^, ^31^ and inadequate glucose transport through the CEP may be an underlying factor in disc degeneration.^3^ While the quantity of the CEP matrix plays an important role in glucose transport, little is known about the effects of matrix cross‐linking, such as the amount of non‐enzymatic cross‐links or AGEs. To address this, we measured glucose uptake in intact human cadaveric CEP tissues from moderately degenerated discs that had undergone in vitro ribosylation to increase AGE formation. Our results show that increased AGE concentrations in the CEP coincided with lower glucose uptake. The ribosylation protocol that we used increased AGE concentrations in the CEP by 131%–179% (Figure 1A). For comparison, the magnitude of those differences is comparable to the AGE accumulation that would be expected to occur with 6.9–17.9 years of natural aging (based on the observed rate of pentosidine accumulation in human intervertebral discs^9^, ^23^, ^32^). Importantly, AGE accumulation had small but significant effects on glucose uptake: on an individual basis, ribosylation reduced glucose partition coefficients in each CEP by 10.7%, on average, compared with donor‐ and site‐matched CEP tissues that did not undergo ribosylation (Figure 2). Those reductions in glucose uptake were observed in the absence of any individual or group differences in CEP characteristics known to independently impact solute uptake, including CEP porosity (*p* = 0.89), hydration (*p* = 0.55), thickness (*p* = 0.99), sGAG content (*p* = 0.47), or collagen content (*p* = 0.61). To investigate mechanisms through which AGE accumulation lowered glucose uptake, we measured the charge density in the CEP matrix using equilibrium partitioning of the CA^4+^ cationic contrast agent with micro‐CT. At equilibrium of contrast enhancement, that is, equilibrium of CA^4+^ diffusion due to electrostatic interactions, mean X‐ray attenuation was 11.9% lower in the CEP tissues that had undergone ribosylation, indicating the CEP matrix was less negatively charged. Solute transport in cartilaginous tissues is known to depend on matrix porosity and fixed charge density, because those characteristics affect the amount of pore space available to the solutes and the extent of electrostatic interactions between fixed charges on the sGAG molecules and the mobile free ions.^33^, ^34^ Our new results extend those findings by demonstrating that non‐enzymatic glycation impacts glucose transport in the CEP independently of matrix porosity or sGAG content, and the effects may be mediated by alterations to matrix charge density.

Our finding that ribosylation lowered glucose uptake in human CEP tissues provides mechanistic insight into correlations observed in previous studies. Specifically, we found that 376‐Da fluorescein uptake was 19% lower in human CEP tissues with high AGE concentrations compared with low AGE concentrations,^7^ but the reason was unclear. Here, in vitro ribosylation enabled us to independently control AGE concentrations and, thereby, quantify the unique effects of AGEs. Our results show that ribosylation for 9 days, which increased AGE concentrations in the CEP by 179% on average (Figure 1A), was associated with 10.7% lower glucose partition coefficients on an individual basis (Figure 2). On the other hand, glucose partition coefficients were nearly 30% lower in the CEP samples with the lowest porosity compared with CEP samples with the highest porosity (Figure S2). Likewise, we previously found that enzymatically reducing sGAG content in the CEP by just 13% was associated with a 19% increase in 376‐Da fluorescein uptake.^7^ Taken together, our results imply that while AGE concentrations are important on an individual level, the effects of inter‐CEP differences in AGEs on glucose transport may be obscured by the effects of inter‐CEP differences in porosity or sGAG content, which play a larger role. Nevertheless, AGE accumulation occurs with aging^9^ and is accelerated by diabetes^12^, ^35^ and diet,^10^, ^36^ and our findings suggest a potential mechanism by which those changes could impact disc nutrition.

To our knowledge, this study is the first to use CA^4+^ to probe the electrostatic effects of AGEs on matrix charge density. This extends the utility of contrast‐enhanced micro‐CT with CA^4+^ in the study of disc degeneration beyond prior work assessing sGAG content. CA^4+^ binds to the negatively charged sGAG chains,^18^, ^19^, ^20^, ^37^ and for this reason, CA^4+^ uptake provides an excellent model system to investigate reactions that alter the fixed charge density of the matrix. The lower X‐ray attenuation post‐contrast enhancement measured in the CEP tissues that had undergone ribosylation indicates a less negatively charged CEP matrix. This is consistent with prior studies showing that increased cross‐link formation by ribosylation hinders interactions between aggrecan and hyaluronan^9^, ^38^ and can also result in the disassembly and impaired function of link protein‐stabilized aggregates,^39^ thereby lowering fixed charge density of the sGAG matrix. On the other hand, ribosylation appears to induce a loss of positive charge in type I collagen,^17^, ^40^ which would be expected to increase cation uptake. Because the X‐ray attenuation was ultimately lower in the ribosylated CEP samples, we conclude that CA^4+^ may be more sensitive to the effects of AGEs on the sGAG matrix than to the effects on the collagen matrix. Moreover, we note that differences in glucose partition coefficients (10.7%) and mean X‐ray attenuation (11.9%) between ribosylated CEP samples and their donor‐ and site‐matched controls were similar in magnitude, which further supports the interpretation that AGE accumulation lowers glucose uptake in the CEP primarily via electrostatic mechanisms.

We used a two‐bath method to measure the equilibrium partition coefficients of glucose (180 Da) in the CEP. Although we are unaware of prior studies that measured glucose partition coefficients in the CEP, the partition coefficients in the control samples (0.85 ± 0.14) were in the range of values reported for other cartilaginous tissues, which supports the external validity of the measurements. Specifically, Torzilli et al.^41^ measured glucose partition coefficients in immature (0.72 ± 0.15) and mature (0.67 ± 0.14) bovine articular cartilage. Notwithstanding, partition coefficients depend on solute charge and molecular weight,^42^ so additional studies are necessary to understand the effects of AGEs on the uptake of other solutes relevant to disc nutrition.

This study had some limitations. Because the biochemical assays were incompatible with the CA^4+^ experiments, it was not possible to perform all of the biochemical, solute uptake, and contrast‐enhanced micro‐CT measurements on the same samples. To minimize potential confounders, the CEP tissues for the different experiments were harvested from the same levels and donors. A related technical limitation is that the relatively large number of samples required to perform all the experiments necessitated the use of small (4‐mm diameter) punches, and the high surface‐to‐volume ratio of the samples may explain why some of the partition coefficients had values >1. A second limitation was that AGE accumulation was increased artificially using in vitro ribosylation, and the effects of in vivo cross‐link accumulation, including the effects of stabilizing enzymatic cross‐links, are unknown and warrant future study. Finally, we measured glucose partition coefficients under free‐swelling conditions, and compressive loading of the cartilage could lower the partition coefficients^25^ and may also depend on the extent of non‐enzymatic glycation. Although we are not aware of any studies that measured the effects of AGEs on CEP mechanical behavior specifically, the finding that non‐enzymatic glycation affected energy dissipation and viscoelastic properties of disc tissues^16^, ^43^ suggests that AGE accumulation may likewise impact CEP mechanical behavior and strain‐dependent transport properties too. Nevertheless, we expect the present data to be the representative of the relative effects of AGE accumulation on glucose uptake in human CEP tissues.

## 5 CONCLUSION

In summary, we found that AGE accumulation in the CEP had a small but significant effect on glucose uptake in human cadaveric CEP tissues. Specifically, ribosylation reduced glucose partition coefficients in the CEP tissues by 10.7% compared with donor‐ and site‐matched control CEP tissues, and those differences in glucose uptake were observed in the absence of any individual or group differences in characteristics of the CEP that reflect matrix quantity. In complementary experiments using contrast‐enhanced micro‐CT with a cationic contrast agent, we found that mean X‐ray attenuation was 11.9% lower in the CEP tissues that had undergone ribosylation, implying the CEP matrix was less negatively charged. We conclude that non‐enzymatic glycation impacts glucose transport in the CEP independently of matrix porosity or sGAG content and that effects may be mediated by alterations to matrix charge density. These findings suggest a potential mechanism by which AGE accumulation in the disc with aging and disease could impact disc nutrition.

## AUTHOR CONTRIBUTIONS

*Conception and design of the work*: Jae‐Young Jung, Shannon C. Timmons, Tristan Maerz, and Aaron Fields. *Acquisition and analysis*: Jae‐Young Jung, Mohamed Habib, Luke J. Morrissette, Shannon C. Timmons, Tristan Maerz, and Aaron Fields. *Interpretation of data for the work*: Jae‐Young Jung, Mohamed Habib, Shannon C. Timmons, Tristan Maerz, and Aaron Fields. All authors drafted or critically revised the manuscript for important intellectual content. All authors approved of the final version to be published and agree to be accountable for all aspects of the work in ensuring that questions related to the accuracy or integrity of any part of the work are appropriately investigated and resolved.

## CONFLICT OF INTEREST STATEMENT

This research was funded by grants from the NIH that were paid to the institution. The authors declare no conflicts of interest.

## Supporting information


**Data S1.** Supporting information.
