# Supplementary material for: Non‐enzymatic glycation reduces glucose transport in the human cartilage endplate independently of matrix porosity or proteoglycan content
Source: JOR Spine. 2023 Oct 24;7(1):e1297. doi: 10.1002/jsp2.1297 (PMC10782066; doi:10.1002/jsp2.1297)
Supplement: Supplementary file 1 — Data S1. Supporting information. [file JSP2-7-e1297-s001.docx]

**SUPPORTING INFORMATION**

**Non-enzymatic glycation reduces glucose transport in the human cartilage endplate independently of matrix porosity or proteoglycan content**

**Jae-Young Jung^1^, Mohamed Habib^1^, Luke J. Morrissette^2^, Shannon C. Timmons^2^,**

**Tristan Maerz^3^, Aaron Fields^1,*^**

^1^Department of Orthopaedic Surgery, University of California, San Francisco, CA 94143

^2^Department of Natural Sciences, Lawrence Technological University, Southfield, MI 48075

^3^Departments of Orthopaedic Surgery and Biomedical Engineering,

University of Michigan, Ann Arbor, MI 48109

*Corresponding Author: Aaron J. Fields; Phone: 415-476-0960; Email: aaron.fields@ucsf.edu

**TABLE OF CONTENTS**

1. General Methods………………………………………………………………………………………………S2
2. Synthesis and Characterization of CA^4+^………………………………………………………………...S2-S4
3. Preparation of the Chloride Salt of CA^4+^………………………………………………............................. S4
4. A relationship between glucose partition coefficient and CEP matrix porosity....................................S5
5. References………………………………………………………………………………...............................S5
6. **General Methods**

All chemicals were purchased commercially and used without further purification. Analytical thin-layer chromatography was performed on polyester-backed TLC plates pre-coated with silica gel containing a fluorescent indicator (200 μm layer thickness, 5-17 μm particle size). Compounds on TLC plates were visualized using a shortwave UV lamp (254 nm). Nuclear magnetic resonance data was recorded using an Agilent MR-400 MHz spectrometer in the Lumigen Instrument Center at Wayne State University. Chemical shifts are reported in parts per million (ppm) and were referenced to the DMSO solvent peak at 2.50 ppm. High-resolution mass spectra were recorded using a Thermo Scientific LTQ Orbitrap XL™ mass spectrometer in the Lumigen Instrument Center at Wayne State University.

1. **Synthesis and Characterization of CA^4+^**

The synthetic protocols described herein were adapted from those previously published by Joshi *et al.* and Newton *et al*.^S1-S3^ The synthetic scheme used to prepare the cationic contrast agent CA^4+^ is shown in Figure S1.

**Figure S1.** Synthetic scheme used to prepare cationic contrast agent CA^4+^

- 1. **Synthesis of 5-amino-2,4,6-triiodoisophthaloyl chloride (1)**

Commercially available 5-amino-2,4,6-triiodoisophthalic acid (10.0 g, 17.9 mmol), thionyl chloride (40.0 mL, 551 mmol), and pyridine (py, 3.25 mL, 40 mmol) were combined in a round-bottomed flask. The resulting reaction mixture was stirred at room temperature for 24 hours. During this time period, all of the 5-amino-2,4,6-triiodoisophthalic reactant dissolved, resulting in a yellow-colored reaction mixture. After 24 hours, TLC revealed the complete consumption of 5-amino-2,4,6-triiodoisophthalic acid. Excess thionyl chloride was removed via two rounds of rotary evaporation, diluting with ethyl acetate between rounds. The resulting yellow solid was dissolved in ethyl acetate (300 mL), washed with a 1:1 saturated aqueous NaCl/saturated aqueous NaHCO_3_ solution (3 x 300 mL), and dried using anhydrous MgSO_4_. This organic layer was subsequently concentrated under reduced pressure to afford a dark amber solid product (10.5 g, 17.6 mmol, 96% yield, R_f_ = 0.54 using 1:2 ethyl acetate/hexane as an eluent). Compound **1** was stored in a vacuum desiccator until it was used in the next reaction without further purification or characterization.

- 1. **Synthesis of tetrakis(acyl chloride) contrast agent (2)**

Compound **1** (14.2 g, 23.9 mmol), 5 Å pelleted molecular sieves (~10), and a magnetic stir bar were placed in a round-bottomed flask fitted with a septum. Following the introduction of a nitrogen atmosphere, anhydrous tetrahydrofuran (THF, 120 mL) was added to the flask, which was swirled and stirred for 15 minutes to promote dissolution. Malonyl chloride (1.2 mL, 12.0 mmol) was subsequently added in a dropwise fashion and the resulting reaction mixture was stirred at room temperature under a nitrogen atmosphere for 24 hours. After 24 hours, TLC revealed the formation of a new compound with a lower R_f_. *n*-Hexane (485 mL) was added to the reaction mixture to precipitate the product. After storing the resulting solution in a -20 °C freezer overnight, the precipitated product was collected via suction filtration as a pearl white solid (11.0 g, 8.75 mmol, 73% yield, R_f_ = 0.54 using 1:1 ethyl acetate/hexane as an eluent). Compound **2** was stored in a vacuum desiccator until it was used in the next reaction without further purification or characterization.

- 1. **Synthesis of tetrakis(Boc-protected) contrast agent (3)**

Compound **2** (9.5 g, 7.5 mmol) and a magnetic stir bar were placed in a round-bottomed flask fitted with a septum. Following the introduction of a nitrogen atmosphere, anhydrous *N,N*-dimethylacetamide (DMA, 75 mL) and triethylamine (TEA, 8.6 mL, 62 mmol) were added to the flask. In a separate round-bottomed flask, mono-Boc-protected ethylenediamine (7.2 mL, 45 mmol) was dissolved in anhydrous DMA (13 mL) under a nitrogen atmosphere. This solution was added dropwise to the initial flask and the resulting reaction mixture was stirred at room temperature for 24 hours. After this time period, TLC revealed the formation of the compound **3** product with an R_f_ of 0. Ethyl acetate (104 mL) and *n*-hexane (300 mL) were added to the reaction mixture to precipitate the product. After storing the resulting solution in a -20 °C freezer overnight, the precipitated product was collected via suction filtration and dried under high vacuum to afford a white solid (7.68 g, 4.38 mmol, 58% yield). Compound **3** was stored in a vacuum desiccator until it was used in the next reaction. ^1^H NMR (400 MHz, DMSO-d_6_, δ): 10.20 (s, 2H, N*H*C(O)CH_2_C(O)N*H*), 8.62 (m, 4H, C(O)N*H*CH_2_), 6.75 (br s, 4H, N*H*C(O)OC(CH_3_)_3_), 3.51 (s, 2H, C(O)C*H_2_*C(O)), 3.22 (br m, 8H, C*H_2_*), 3.16 (br m, 8H, C*H_2_*), 1.38 (s, 36H, C*H_3_*); HRMS (ESI-TOF) *m/z*: [M+Na]^+^ calcd for C_47_H_64_O_14_N_10_I_6_Na, 1776.8764; found, 1776.8777.

- 1. **Synthesis of tetrakis(amino) contrast agent, trifluoroacetate salt (CA^4+^)**

Compound **3** (7.20 g, 4.10 mmol) and a magnetic stir bar were placed in a round-bottomed flask fitted with a septum. Following the introduction of a nitrogen atmosphere, anhydrous dichloromethane (DCM, 44 mL) and trifluoroacetic acid (TFA, 44 mL) were added to the flask. The resulting reaction mixture was stirred at room temperature for 24 hours. After this time period, the reaction mixture was concentrated under reduced pressure and the product was precipitated from the resulting syrup using ethyl acetate (260 mL). After storing the resulting solution in a -20 °C freezer overnight, the precipitated product was collected via suction filtration and dried under high vacuum to afford a white solid (6.41 g). The **CA^4+^** contrast agent was stored in a vacuum desiccator. ^1^H NMR (400 MHz, DMSO-d_6_, δ): 10.27 (s, 2H, N*H*C(O)CH_2_C(O)N*H*), 8.80 (m, 4H, C(O)N*H*CH_2_), 8.25 (br s, 8H, N*H*_2_), 3.52 (s, 2H, C(O)C*H_2_*C(O)), 3.47 (br m, 8H, C*H_2_*), 2.99 (br m, 8H, C*H_2_*); HRMS (ESI-TOF) *m/z*: [M+H]^+^ calcd for C_27_H_33_O_6_N_10_I_6_, 1354.6847; found, 1354.6850.

1. **Preparation of the Chloride Salt of CA^4+^**

The trifluoroacetate salt of **CA^4+^** was dissolved in a minimal amount of distilled water and the pH was adjusted to 1.0 using 3 M aqueous HCl. The resulting solution was lyophilized to yield the corresponding chloride salt as an off-white solid (4.63 g, 3.09 mmol, 75% yield). The chloride salt of **CA^4+^** was stored in a vacuum desiccator until further use in imaging experiments.

1. **Relationship between glucose partition coefficient and CEP matrix porosity**


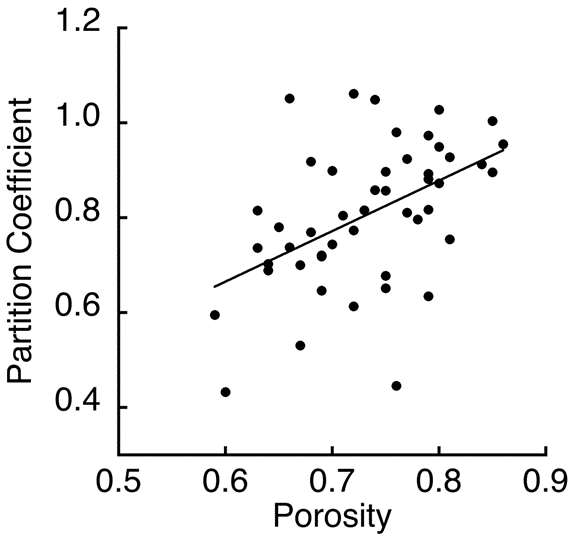


**Figure S2.** Partition coefficient of glucose (180 Da) in human cadaveric CEP tissues was significantly associated with CEP matrix porosity (*r^2^* = 0.24, *p* = 0.0005). n = 48 CEP samples from 12 cadavers.

1. **References**

**S1.** Joshi, N. S.; Bansal, P. N.; Stewart, R.; Snyder, B. D.; Grinstaff, M. W. Effect of contrast agent charge on visualization of articular cartilage using computed tomography: Exploiting electrostatic interactions for improved sensitivity. *J. Am. Chem. Soc.* **2009**, *131*, 13234-13235.

**S2.** Newton, M. D.; Hartner, S. E.; Timmons, S.; Delaney, N. D.; Pirrone, M. G.; Baker, K. C.; Maerz, T. Contrast-enhanced µCT of the intervertebral disc: A comparison of anionic and cationic contrast agents for biochemical and morphological characterization. *J. Orthop. Res.* **2017**, *35*, 1067-1075.

**S3.** Newton, M. D.; Hartner, S. E.; Gawronski, K.; Davenport, E. J.; Timmons, S. C.; Baker, K. C.; Maerz, T. Nondestructive, indirect assessment of the biomechanical properties of the rat intervertebral disc using contrast-enhanced µCT. *J. Orthop. Res.* **2018**, *36*, 2030-2038.
